# The Mixed-Lineage Kinase DLK Is a Key Regulator of 3T3-L1 Adipocyte Differentiation

**DOI:** 10.1371/journal.pone.0004743

**Published:** 2009-03-09

**Authors:** Jean-Philippe Couture, Alex Daviau, Julie Fradette, Richard Blouin

**Affiliations:** 1 Département de biologie, Faculté des sciences, Université de Sherbrooke, Sherbrooke, Québec, Canada; 2 Laboratoire d'Organogenèse Expérimentale, Centre Hospitalier Affilié Universitaire de Québec, Hôpital du Saint-Sacrement, Québec, Canada; Brunel University, United Kingdom

## Abstract

**Background:**

The mixed-lineage kinase (MLK) family member DLK has been proposed to serve as a regulator of differentiation in various cell types; however, its role in adipogenesis has not been investigated. In this study, we used the 3T3-L1 preadipocyte cell line as a model to examine the function of DLK in adipocyte differentiation.

**Methods and Findings:**

Immunoblot analyses and kinase assays performed on 3T3-L1 cells showed that the expression and activity of DLK substantially increase as differentiation occurs. Interestingly, DLK appears crucial for differentiation since its depletion by RNA interference impairs lipid accumulation as well as expression of the master regulators of adipogenesis C/EBPα and PPARγ2 at both the mRNA and protein levels. In contrast, neither the expression nor the DNA binding activity of C/EBPβ, an activator for C/EBPα and PPARγ, is affected by DLK loss.

**Conclusions:**

Taken together, these results suggest that DLK is important for expression of mature adipocyte markers and that its action most likely takes place via regulation of C/EBPβ transcriptional activity and/or initiation of C/EBPα and PPARγ2 gene transcription.

## Introduction

Adipose tissue, the main organ for energy storage and expenditure, secretes a variety of factors that maintain normal body metabolism [1]. Its accumulation, however, can result in obesity, which is a major risk factor for diseases such as diabetes and hypertension [2]–[6]. Change in adipose mass can arise from an increase in the size and the number of fat cells or adipocytes, the latter being accomplished by the proliferation of preadipocytes and their subsequent differentiation into mature adipocytes [7].

Due to the availability of preadipocyte cell lines such as 3T3-L1 and 3T3-F422A [8], which can be efficiently induced to undergo terminal differentiation when exposed to the appropriate adipogenic hormones, considerable progress in our understanding of adipocyte biology has been achieved in the past few years. Indeed, the process of adipocyte differentiation is governed by a tightly regulated cascade of transcription factors that are either activated or repressed at specific times during differentiation [9]–[11]. These variations in expression or activation lead to differential gene expression that will eventually guide precursor cells through their differentiation in adipocytes. Important members of this genetic cascade are the CCAAT/enhancer binding proteins (C/EBP) family members C/EBPβ and C/EBPδ, which are highly expressed early during differentiation [12]. C/EBPβ and C/EBPδ then elicit the expression of C/EBPα and the PPARγ (Peroxisome proliferator-activated receptor γ) isoform PPARγ2, two transcription factors working in a cooperative manner to promote the expression of various adipocyte-specific genes [13]. Their ability to control the expression of genes responsible for glucose trafficking and mature adipocyte metabolism [11] indeed place them as key factors of adipocyte differentiation.

Besides transcription factors, a variety of extracellular and intracellular signaling molecules are also known to play key roles in adipocyte differentiation [11], [14]–[17]. Of particular interest to this study is the demonstration that mitogen-activated protein kinases (MAPKs) [18], which include extracellular signal-regulated kinases (ERKs), p38 kinases and c-Jun N-terminal kinases (JNKs), modulate either positively or negatively adipogenesis as a result of their ability to regulate the proadipogenic transcription factors C/EBPβ and PPARγ [19], [20]. Recently, it has also been shown that MLK3, a JNK activator belonging to the mixed-lineage kinase (MLK) subgroup of MAPK kinase kinase (MAPKKK), plays a role in adipocyte differentiation [21]. Support for this notion derives from the observation that the expression and phosphorylation of C/EBPα and C/EBPβ were significantly increased at early times during differentiation of mouse embryonic fibroblasts (MEF) deficient in *Mlk3* (MLK3^−/−^). Furthermore, it was found that MLK3^−/−^ cells accumulate more lipids than wild-type MEF and that overexpression of MLK3 in these cells inhibited adipogenic differentiation.

In contrast to MLK3, which is widely expressed in many tissues [21], the MLK family member dual leucine zipper-bearing kinase (DLK) exhibits a more restricted pattern of expression [22], [23]. During development, expression of DLK mRNA has been primarily detected in neuronal tissues such as brain and spinal ganglion, as well as in the epithelia of the skin, intestine, pancreas, and kidney [22]. In all these tissues, the expression of DLK mRNA increases with development and correlates with areas undergoing terminal cell differentiation. Consistent with a causal role for DLK in differentiation, ectopic expression of DLK in normal human keratinocytes promotes their terminal differentation, as evidenced by up-regulation of filaggrin, DNA fragmentation and activation of transglutaminases [24]. Based on these observations, it thus appears likely that DLK may fulfill specific signaling functions required in either the induction or the maintenance of the differentiated state for a wide variety of cell populations. In keeping with this hypothesis, we demonstrate herein that DLK is expressed in mouse adipose tissue and that its expression dramatically increases during adipogenic differentiation of 3T3-L1 cells. In addition, our results show that 3T3-L1 cells depleted in DLK by specific shRNA failed to accumulate lipids or express C/EBPα and the adipocyte-specific PPARγ2 isoform in response to adipogenic stimuli. Finally, we provide evidence that DLK depletion does not perturb the ability of C/EBPβ to bind to the *c/ebpα* and *pparγ2* promoters, indicating that DLK acts upstream of the master adipogenesis regulators C/EBPα and PPARγ2 but downstream of their activator C/EBPβ.

## Results

### DLK is expressed in adipose tissue and differentiating adipocytes

**Figure 1:**
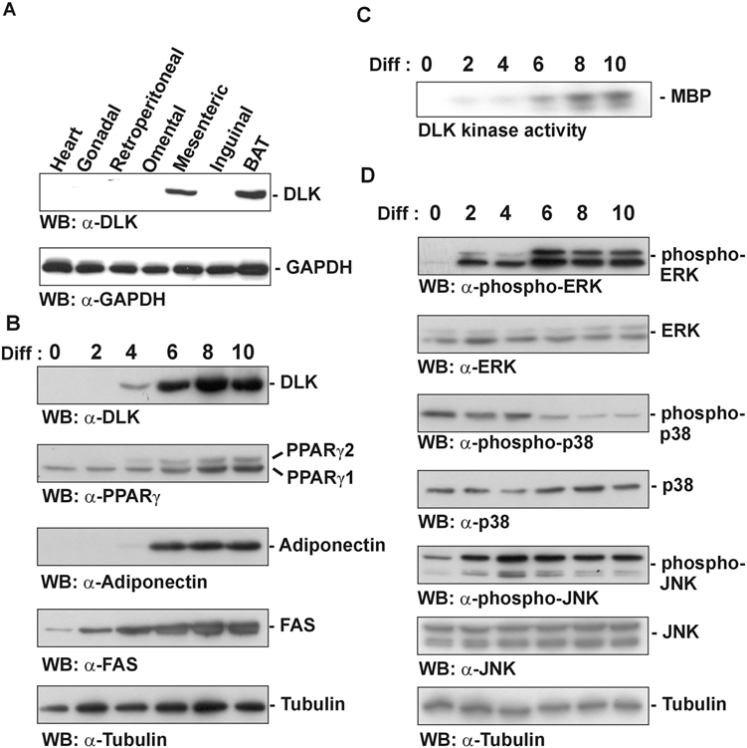
Up-regulation of DLK expression and activity during 3T3-L1 adipocyte differentiation. (A) Homogenates of mouse heart, white adipose tissue from various depots (gonadal, retroperitoneal, omental, mesenteric and inguinal) and brown adipose tissue (BAT) were subjected to Western blot analysis with specific antibodies against DLK or GAPDH. (B) and (D) 3T3-L1 cells induced to differentiate for 2, 4, 6, 8 and 10 days were lysed and subjected to immunoblotting analysis with antibodies to DLK, adiponectin, fatty acid synthase (FAS), PPARγ, ERK, phospho-ERK, p38, phospho-p38, JNK, phospho-JNK and tubulin as the loading control. The antibody raised against PPARγ reveals the presence of both PPARγ isoforms, PPARγ1 and PPARγ2. (C) 3T3-L1 cells induced to differentiate for the indicated times were lysed for immunoprecipitation analysis with anti-DLK antibody and subjected to an immunocomplex kinase assay using myelin basic protein (MBP) as a substrate. *Diff: Days of differentiation*. *WB: Western Blot*.

Because DLK is expressed in a tissue-specific manner [22]–[24], it has been proposed to serve as a regulator of differentiation. To further characterize the function of DLK, we decided to investigate whether this protein is involved in adipocyte differentiation. This issue was first addressed by examining the protein levels of DLK in the adipose organ, which is composed in mammals of white and brown adipose tissues organized in various subcutaneous or visceral depots. White adipose tissue is specialized for lipid storage, whereas brown adipose tissue generates body heat [25]. Immunoblot analysis of various white (gonadal, retroperitoneal, omental, mesenteric and inguinal) and brown (intrascapular) adipose depots showed that DLK is expressed to high levels in both mesenteric white adipose and brown adipose tissue. In comparison, heart as well as gonadal, retroperitoneal, omental and inguinal white adipose tissue depots expressed barely detectable levels of DLK (Fig. 1 A). The presence of DLK in differentiating adipocytes was then assessed in a widely used model of adipogenesis, 3T3-L1 preadipocytes [8], that were induced to differentiate for different periods of time (2, 4, 6, 8 or 10 days). Differentiation of 3T3-L1 cells was confirmed by blotting total cell lysates with antibodies directed against the two PPARγ isoforms, PPARγ1 and PPARγ2, and the mature adipocyte markers adiponectin and fatty acid synthase (FAS) (Fig. 1B). Interestingly, DLK expression in differentiating cells increased gradually until reaching a plateau from day 6 to day 10 of differentiation (Fig. 1B), and this increase paralleled that seen with the protein levels of PPARγ1, PPARγ2 and adiponectin, . Moreover, an immunocomplex kinase assay showed that DLK is active during differentiation of 3T3-L1 (Fig. 1C), reflecting the accumulation of DLK detected by immunobloting.

To explore the presence and activation of the MAPKs ERK, JNK and p38 in differentiating adipocytes, immunoblot analyses with antibodies specific to the phosphorylated, activated forms of these proteins were also performed. As shown in Fig. 1D, ERK was inactive in undifferentiated cells but became active from day 2 of differentiation onward. On the other hand, our results also demonstrated that JNK and p38 exist as constitutively phosphorylated proteins in 3T3-L1 preadipocytes, and upon differentiation of the cells, p38 activity progressively decreased while JNK activity increased to reach a maximum at day 4. Immunoblots processed in parallel with antibodies insensitive to the phosphorylation state of these MAPKs demonstrated that there was no apparent difference in the expression of either ERK, p38 or JNK during adipocyte differentiation, suggesting that their activity are positively or negatively regulated by the differentiation inducers.

### DLK is required for lipid accumulation in 3T3-L1 cells

**Figure 2:**
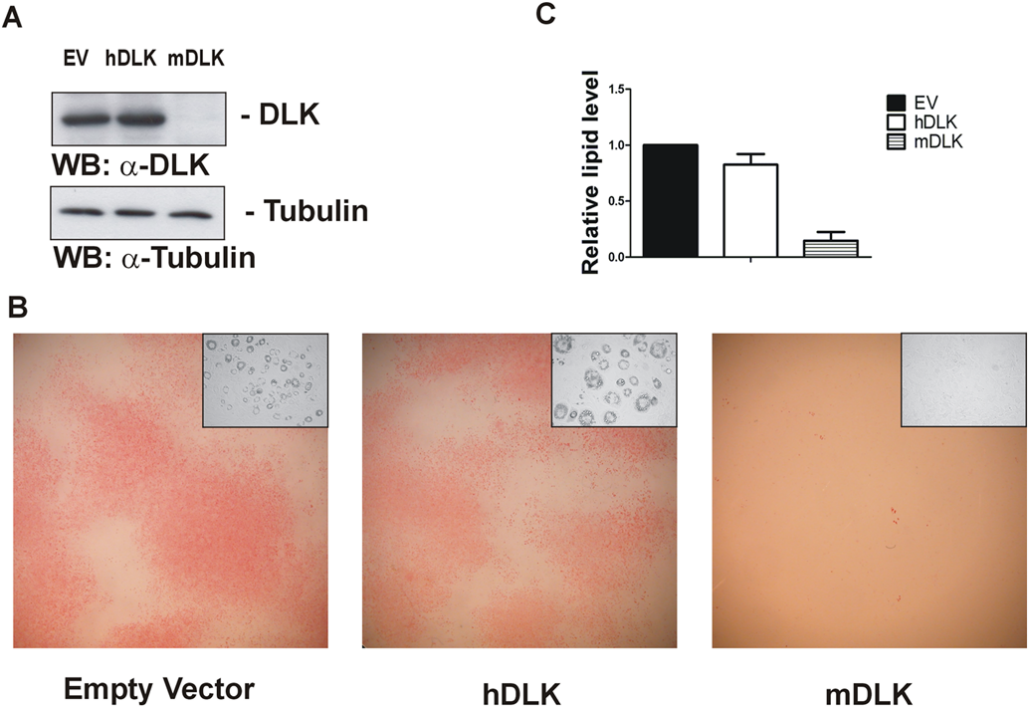
DLK depletion in 3T3-L1 impairs lipid accumulation in response to differentiation inducers. 3T3-L1 cells infected with an empty lentiviral vector (EV) or with a lentivirus expressing human (hDLK) or mouse (mDLK) DLK shRNA were induced to differentiate for 6 days. After differentiation, cells were subjected to Western blot analysis with an antibody directed against DLK (A) or stained for lipids with ORO and photographed (B). As a control for protein loading, immunoblots were probed in parallel with an antibody specific for tubulin. The inset at the upper right corner of each photograph represents unstained cells at a 20× magnification. (C) Lipids extracted from ORO-stained cells were quantified by spectrophotometry at 520 nm. The data represent the mean±SEM of three independent experiments, relative to control EV-infected cells.

To further characterize the role of DLK in adipogenesis, we silenced its expression in 3T3-L1 preadipocytes by RNA interference and stained cells for lipids with Oil Red O (ORO) at day 6 of differentiation. Knockdown of DLK was accomplished by infecting cells with a lentiviral vector carrying a short hairpin RNA (shRNA) that targets mouse DLK mRNA (mDLK). To exclude potential nonspecific effects, cells were also infected with an empty lentiviral vector (EV) or a lentiviral vector expressing a human DLK shRNA (hDLK). In some experiments, the vector for hDLK had slight effect on DLK expression in 3T3-L1 cells, but this was considered negligible when compared to cells infected with the sh-mDLK lentivirus. As depicted in Figure 2A, the sh-mDLK construct abolished almost completely DLK protein expression, whereas the empty or sh-hDLK vector had no such effect. The intracellular level of tubulin was also unaffected by any of these constructs. ORO staining clearly demonstrated that there was no obvious accumulation of lipids in DLK-depleted cells, as opposed to control cells (Fig. 2B). Lack of lipid accumulation could not be attributed to cell mortality, as preadipocyte-shaped undifferentiated cells were still visible after 6 days of differentiation (upper right corner, Fig 2B). Spectrophotometric analysis of the extracted neutral lipids confirmed that DLK-depleted cells were devoid of lipids when compared to cells infected with the control lentiviruses (Fig. 2C).

### Loss of DLK impairs expression of the master regulators of adipogenesis C/EBPα and PPARγ2

**Figure 3:**
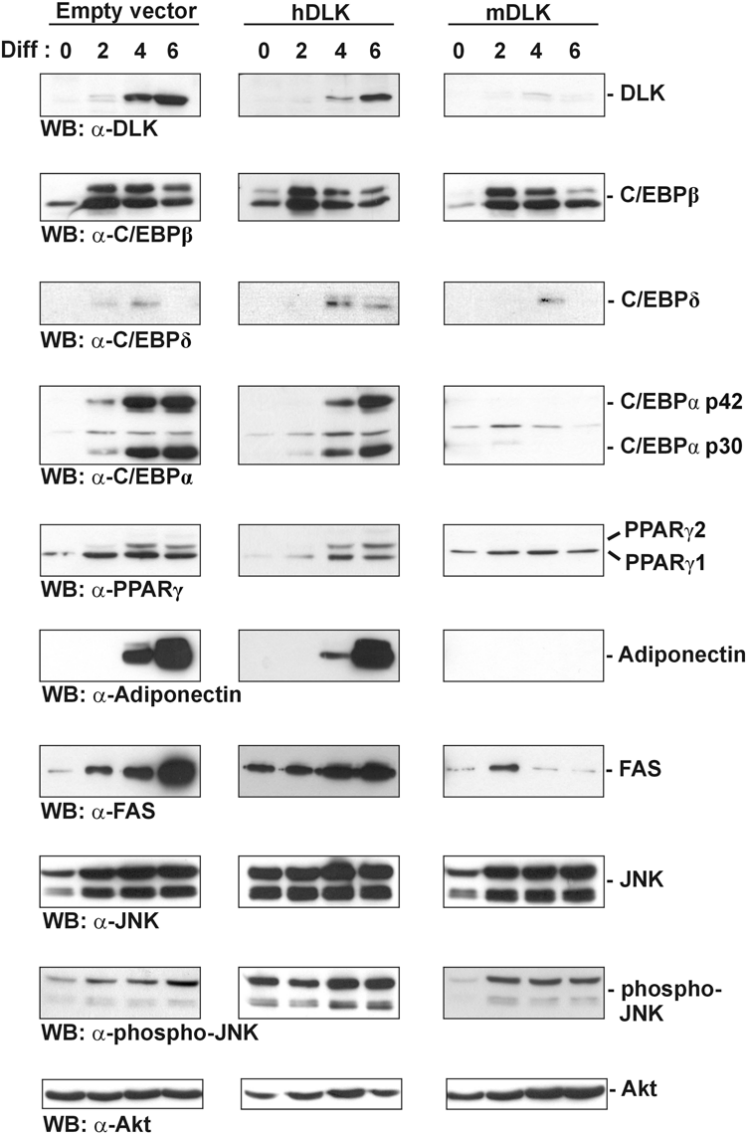
Loss of DLK in 3T3-L1 cells prevents expression of C/EBPα, PPARγ, adiponectin and fatty acid synthase proteins but not that of C/EBPβ. 3T3-L1 cells infected with an empty lentivirus, a lentivirus expressing human DLK shRNA (hDLK) or a lentivirus expressing mouse DLK shRNA (mDLK) were induced to differentiate for the indicated times. After differentiation, cells were subjected to Western blot analysis with specific antibodies against DLK, C/EBPβ, C/EBPδ, C/EBPα, PPARγ, adiponectin, fatty acid synthase (FAS), phospho-JNK, JNK and Akt as the loading control.

As a result of the finding that DLK depletion was inhibitory for accumulation of lipids in 3T3-L1 cells, we next sought to investigate whether the absence of DLK could disrupt the transcription factor cascade that mediates adipocyte differentiation. For this purpose, 3T3-L1 cells were infected with the empty lentivirus or lentiviruses expressing mouse or human DLK shRNA and induced to differentiate for 6 days. The expression levels of four transcription factors known to be involved in adipogenesis, namely C/EBPδ, C/EBPβ, C/EBPα and PPARγ [26] were measured every two days by Western blotting with specific antibodies. As shown in Figure 3, cells infected with the sh-mDLK lentivirus showed a marked reduction of DLK protein expression compared with the control. This depletion of DLK proteins had no effect on the expression of C/EBPβ and C/EBPδ, which are induced early in the differentiation process [12]. In fact, both factors were indeed detectable at day 2 and 4 of differentiation, respectively, and decreased afterwards (Fig. 3). Interestingly, immunoblot analysis also demonstrated that the knockdown of DLK strongly reduced expression of two master regulators of adipogenesis, C/EBPα [27] and the PPARγ2 isoform [28], which were induced as early as day 2 or day 4 of differentiation in cells infected with the control lentiviruses (Fig. 3). Accordingly, expression of the mature adipocyte markers adiponectin and FAS was impaired in DLK-depleted cells compared with cells infected with control lentiviruses. The effect of DLK depletion on C/EBPα and PPARγ2 was not attributable to a change in the subcellular localization of C/EBPβ, which is essential for their expression [29], since the presence of C/EBPβ in the nucleus was confirmed by immunoblot analysis of nuclear and cytoplasmic fractions (data not shown). Moreover, the activity of JNK, which acts as a downstream effector of DLK signaling in various cell types [24], [30], [31], was also not affected by DLK knockdown. Taken together, these data suggest that the decrease of DLK not only prevents the accumulation of lipids, but also disrupts the whole differentiation program of 3T3-L1 by impairing the normal expression of C/EBPα and PPARγ2.

### DLK is required for expression of the C/EBPα, PPARγ, adiponectin and FAS genes

**Figure 4:**
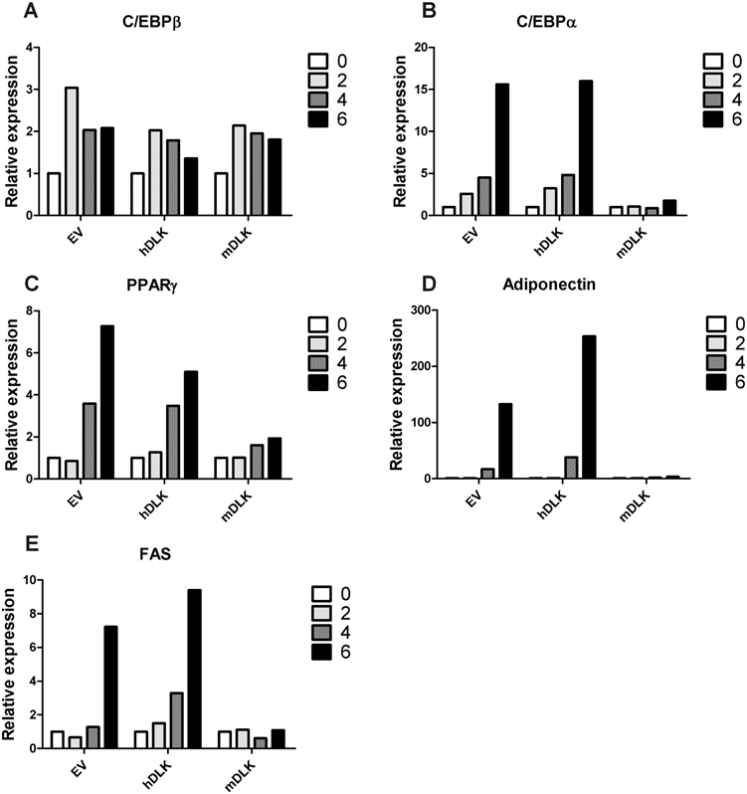
Depletion of DLK in 3T3-L1 cells blocks expression of PPARγ and C/EBPα at the mRNA levels. 3T3-L1 cells infected with an empty lentivirus (EV), a lentivirus expressing human DLK shRNA (hDLK) or a lentivirus expressing mouse DLK shRNA (mDLK) were induced to differentiate, and at the indicated times, total RNA was extracted. C/EBPβ, C/EBPα, PPARγ, adiponectin and FAS mRNA levels were analyzed by quantitative RT-PCR with specific primers. The expression level of each gene was normalized to the level of the 36B4 housekeeping gene. Results are expressed as fold induction of mRNA levels relative to cells harvested on day 0 and they are representative of at least three independent experiments.

Since DLK depletion abrogated the accumulation of C/EBPα, PPARγ, adiponectin and FAS proteins in differentiating 3T3-L1 adipocytes, we next asked whether interruption of DLK signaling would lead to decreased expression of their encoding genes. To do this, we isolated total RNA from control or DLK-depleted cells at day 0, 2, 4 or 6 of differentiation and analyzed the expression of the C/EBPα, PPARγ, adiponectin and FAS genes by quantitative RT-PCR (Fig. 4). For each gene examined during adipocyte differentiation, we observed that the amount of mRNA fluctuated in a pattern similar to that seen at the protein levels (Fig. 3). Hence, in either EV-, hDLK- or mDLK-infected cells, the levels of C/EBPβ mRNA increased at day 2 of differentiation, like its protein counterpart, followed by a slight decrease in more differentiated 3T3-L1 adipocytes (Fig. 4A). However, for C/EBPα, PPARγ, adiponectin and FAS mRNAs (Fig 4B to 4E), which are all induced later in adipogenesis, no increase of their expression levels was observed in mDLK-infected cells relative to control cells. These results indicate that DLK is required for expression of the C/EBPα, PPARγ, adiponectin and FAS genes in differentiating adipocytes.

### DLK depletion does not impair C/EBPβ binding activity *in vivo*

**Figure 5:**
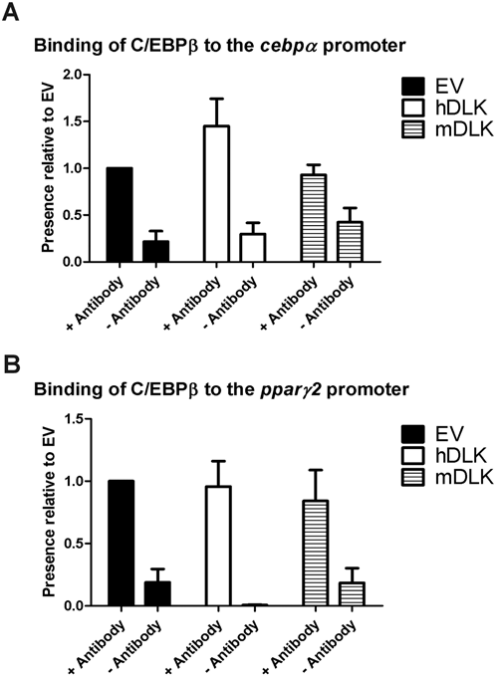
Knockdown of DLK does not interfere with the binding of C/EBPβ to the *cebpα* and *pparγ2* promoters. EV-, hDLK- and mDLK-infected 3T3-L1 cells induced to differentiate for two days were subjected to chromatin immunoprecipitation with an antibody specific to C/EBPβ. The precipitated chromatin was analyzed by quantitative PCR using primers spanning the binding site of C/EBPβ within the *cebpα* and *pparγ2* promoters. The data represent the mean±SEM of three independent experiments, relative to control EV-infected cells.

An important function of C/EBPβ during adipocyte differentiation is to directly activate expression of C/EBPα and PPARγ2 [29], [32]. Based on these data and our results showing that expression of C/EBPβ was not attenuated by DLK depletion, we next investigated by chromatin immunoprecipitation (ChIP) assays the binding activity of endogenous C/EBPβ to the C/EBPα and PPARγ2 promoters in 3T3-L1 cells infected with the different lentiviral constructs. DNA fragments immunoprecipitated by C/EBPβ antibody at day 2 of differentiation, a time window where C/EBPβ expression peaked, were amplified by PCR using primers covering C/EBPβ binding sites within the C/EBPα and PPARγ2 promoters. As shown in Fig. 5, we observed no change in C/EBPβ binding activity at both promoters after DLK depletion, suggesting that loss of DLK does not impair C/EBPβ's ability to stimulate transcription of C/EBPα and PPARγ2 genes.

### Activation of PPARγ1 by rosiglitazone rescues adipocyte differentiation of DLK-depleted 3T3-L1 cells

**Figure 6:**
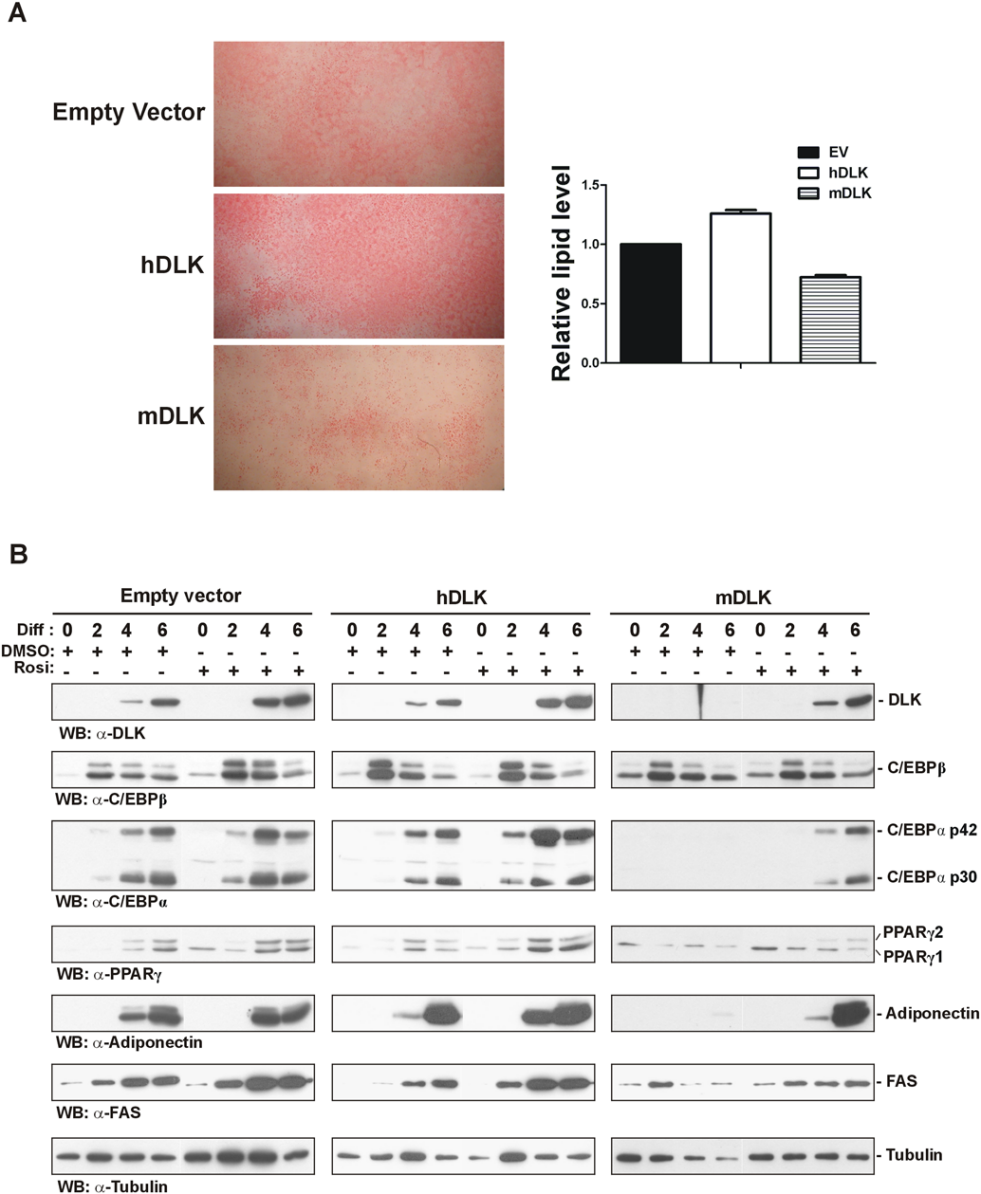
PPARγ activation by rosiglitazone rescues adipocyte differentiation in DLK-depleted 3T3-L1 cells. (A) 3T3-L1 cells infected with an empty lentiviral vector (EV) or with a lentivirus expressing human (hDLK) or mouse (mDLK) DLK shRNA were induced to differentiate for 6 days in the presence of 1 µM rosiglitazone. After differentiation, cells were stained for lipids with ORO and photographed. Lipids extracted from ORO-stained cells were quantified by spectrophotometry at 520 nm. The data represent the mean±SEM of three independent experiments, relative to EV-infected cells. (B) 3T3-L1 cells infected with an empty lentiviral vector (EV) or with a lentivirus expressing human (hDLK) or mouse (mDLK) DLK shRNA were induced to differentiate for 2, 4 or 6 days in the presence of 1 µM rosiglitazone or DMSO (vehicule). After differentiation, cells were subjected to Western blot analysis with antibodies directed against DLK, C/EBPβ, C/EBPα, PPARγ, adiponectin and FAS. As a control for protein loading, immunoblots were probed in parallel with an antibody specific for tubulin.

PPARγ2 is a central regulator of adipogenesis [33], whose expression at the mRNA and protein levels is down-regulated in DLK-depleted 3T3-L1 cells. We therefore investigated whether the inhibitory effect of DLK depletion on adipocyte differentiation was specifically caused by prevention of the expression of PPARγ2 and C/EBPα. To do so, we tested whether rosiglitazone, a well known PPARγ ligand [34], could rescue differentiation of 3T3-L1 cells after DLK knockdown. 3T3-L1 cells were infected with the different lentiviruses and then subjected to the differentiation protocol for 6 days in the presence of rosiglitazone. Addition of rosiglitazone to mDLK-infected cells restored the characteristic lipid accumulation associated with adipocyte differentiation, although not to the extent seen in EV- and hDLK-infected cells (Fig. 6A). Spectrophotometric quantification of the extracted lipids showed that rosiglitazone-treated mDLK-infected cells accumulate approximately 75% of the lipids that are found in control cells (Fig 6A). Rosiglitazone treatment of DLK-depleted cells also rescued, at least in part, the expression of C/EBPα, PPARγ2, adiponectin and FAS, as revealed by immunoblot analyses carried out at day 2, 4 and 6 of differentiation (Fig. 6B). Unexpectedly, an increase in DLK levels was also observed in rosiglitazone-treated control and DLK knockdown adipocytes, suggesting the potential involvement of an activated form of PPARγ in DLK expression. Taken together, these results indicate that rosiglitazone can overcome the inhibitory effect of DLK depletion on adipocyte differentiation and suggest that the loss of DLK principally inhibits the differentiation program of 3T3-L1 cells by preventing the expression of PPARγ2.

## Discussion

Adipogenesis is a complex process governed by a wide range of regulatory proteins, including transcription factors, kinases and hormones. Adding to this complexity, our results identify the MLK family member DLK as a novel regulator of adipocyte differentiation. Immunoblot analyses of various mouse adipose depots indeed demonstrated that DLK was specifically expressed in mesenteric white and brown adipose tissue, suggesting a role for this protein in differentiation and function of particular adipocyte subpopulations. Using the 3T3-L1 preadipocyte cell line as a model, we then showed that the protein level of DLK gradually increased upon differentiation like the adipogenic markers PPARγ2, adiponectin and fatty acid synthase. This increase in DLK expression was paralleled by either an increase or decrease in phosphorylation of the MAPKs ERK, p38 and JNK, which are recognized to have both positive and negative regulatory effects on adipocyte differentiation [20]. Using gene silencing of DLK by RNA interference, we demonstrated that this protein is essential for lipid accumulation and expression of the two master regulators of adipocyte differentiation, C/EBPα and PPARγ2 [27], [33], [35]. In agreement with this result, we found that DLK depletion was accompanied by decreased expression of adiponectin and fatty acid synthase that are downstream genes of C/EBPα, PPARγ [36], [37] and SREBP-1c (sterol regulatory element binding protein 1 c), a transcription factor involved in fatty acid metabolism [38], respectively. This effect was reversed by the PPARγ agonist rosiglitazone, suggesting that the absence of DLK does not impair the capacity of an activated form of PPARγ to promote adipogenesis, and that DLK action takes place upstream of PPARγ. The capacity of ligand-activated PPARγ to rescue adipogenesis in DLK-depleted cells is in agreement with previous work showing the relative contribution of the two different PPARγ isoforms, PPARγ1 and PPARγ2, to adipogenesis. In their study, using engineered 3T3-L1 cells devoid of PPARγ1 and PPARγ2, Ren et al. [28] demonstrated that only ectopic expression of PPARγ2 can induce adipocyte differentiation in the absence of exogenous ligand. Although it is not clear why this difference is observed, Werman and colleagues [35] reported that PPARγ2 contains a constitutive activation function in the N-terminus that is up to 10-fold stronger than that of PPARγ1. Moreover, it has been found that the N-terminus of PPARγ2 binds a small protein, termed PGC-2, itself having adipogenic action [39]. Thus, combined to these findings, our results suggest that the absence of DLK most likely prevents adipocyte conversion of 3T3-L1 cells by impairing the expression of PPARγ2.

Of particular interest was the finding that neither the expression nor the nuclear localization of C/EBPβ was affected by the loss of DLK. This suggests that the early events in adipogenesis such as CREB (cAMP response element binding protein) activation by protein kinase A [40] and its consequent up-regulation of C/EBPβ remain unmodified under these conditions. RNA interference studies also led us to find that the depletion of DLK has no effect on JNK activity during 3T3-L1 adipocyte differentiation. Although surprising, since DLK has been identified as an upstream activator of the JNK pathway [41], this result is not entirely without precedent. Published data from overexpression and RNA interference-mediated knockdown studies in other cell systems, such as COS and NIH 3T3 cells, also support a role for DLK in activation of the p38, ERK and Akt signaling pathways [31], [42]. Therefore, the possibility that an effector other than JNK mediates the action of DLK during adipogenesis in 3T3-L1 cells can not be excluded.

As demonstrated by the results of our RT-qPCR analyses, DLK depletion affects the accumulation of C/EBPα, PPARγ, adiponectin and fatty acid synthase proteins during adipocyte differentiation by directly down-regulating the expression of their encoding genes. Because ChIP assays revealed no difference in C/EBPβ recruitment to the *cebpα* and *pparγ2* promoters before and after DLK depletion, it is tempting to speculate that DLK action in adipogenesis lies between C/EBPβ DNA binding and initiation of *cebpα* and *pparγ2* gene transcription. C/EBPβ binding to the *cebpα* and *pparγ2* promoters without being able to induce their transcription is a naturally occurring process during adipogenesis. Indeed, once induced by adipogenic stimuli, C/EBPβ binds to the *cebpα* and *pparγ2* promoters well before initiation of transcription starts [12]. This is followed by recruitment of the Ini1, Brg1 and Brm subunits of the chromatin-remodelling SWI/SNF complex [43] at the *pparγ2* promoter, which leads to activation of *pparγ2* transcription. The resulting accumulation of PPARγ2 is a prerequisite to the expression of C/EBPα, since active PPARγ2 has the ability to displace a repressive complex composed of mSin3A/histone deacetylase (HDAC)1 from the *cebpα* promoter [29]. Thus, if the SWI/SNF complex is not recruited to the *pparγ2* promoter, neither PPARγ2 nor C/EBPα will be expressed during adipogenesis, a phenomenon similar to what is seen in DLK-depleted 3T3-L1 cells. Our observation that rosiglitazone treatment restores, at least in part, the expression of C/EBPα in DLK-depleted cells is consistent with the idea that PPARγ activation facilitates C/EBPα expression [28]. Thus, it is likely that rosiglitazone-mediated activation of PPARγ1 in DLK-depleted cells is sufficient to displace the repressive mSin3A/HDAC1 complex from the *cebpα* promoter and allow the expression of C/EBPα, which in turn induces PPARγ2, the most potent regulator of adipogenesis [44].

Another potential mechanism by which DLK depletion might decrease PPARγ2 and C/EBPα mRNA levels is by altering phosphorylation of C/EBPβ. This idea is supported by the fact that C/EBPβ has multiple phosphorylation sites, some of which are involved in the regulation of DNA-binding activity [16], while others are key determinants of its transactivation capacity [17]. Of particular interest among them is threonine 188, a consensus phosphorylation site for both ERK and glycogen synthase kinase 3 (GSK3) [16]. Mutation of this threonine to alanine disrupts C/EBPβ's ability to activate C/EBPα expression, but not DNA binding to C/EBP response element within the proximal promoter [17]. The T188A mutation also makes C/EBPβ incapable of inducing adiponectin gene expression, probably as a result of loss of C/EBPα expression. Taken together, these results imply that phosphorylation of C/EBPβ at threonine 188 is critical for transcriptional activity in the context of the C/EBPα promoter. Additional support for a role of phosphorylation in the control of C/EBPβ function also comes from the findings of Roy et al. who recently demonstrated that MLK3 activates C/EBPβ in response to IFN-γ by a mechanism involving decreased phosphorylation of a specific serine residue within transactivation domain [45]. Therefore, even if C/EBPβ binds the *cebpα* and *pparγ2* promoters in DLK-depleted cells as efficiently as in control cells, a change in its phosphorylation state that would impair its function or interaction with transcriptional co-activators is also consistent with our results.

**Figure 7:**
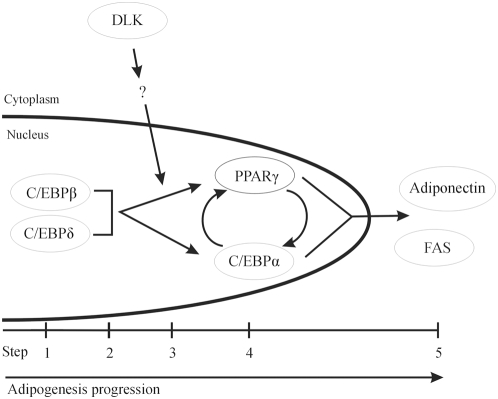
Proposed model for the role of DLK in adipogenesis. Early events in adipogenesis lead to the expression of C/EBPβ and C/EBPδ (step 1), which then bind to the *cebpα* and *pparγ2* promoters (step 2). DLK (step 3) in turn appears to contribute to the subsequent increase in the expression of PPARγ and C/EBPα (step 4) by a mechanism that remains to be identified. The latter two proteins then direct the expression of various adipocyte-specific genes (step 5).

In summary, the results presented in this report identify DLK, a MLK family member, as one of the key regulators of adipocyte differentiation. Although the exact mechanisms by which DLK controls this process remain to be identified, DLK action most likely takes place downstream of C/EBPβ DNA-binding to the *cebpα* and *pparγ2* promoters but upstream of transcription initiation (Fig. 7).

## Materials and Methods

### Chemicals and antibodies

Dexamethasone, 3-isobutyl-1-methylxanthine (IBMX), insulin, protease inhibitors, the mouse monoclonal antibody raised against α-tubulin and all others common reagents were purchased from Sigma-Aldrich Ltd. (Saint Louis, MO, USA). Rosiglitazone was purchased from Cayman Chemicals, via Cedarlane (Burlingont, Ontario, Canada). The rabbit polyclonal antibody raised against DLK was obtained from Abgent (San Diego, CA, USA). The rabbit polyclonal antibodies raised against JNK, phospho-JNK, C/EBPα, C/EBPβ, C/EBPδ and PPARγ were obtained from Santa-Cruz Biotechnology Inc. (Santa Cruz, CA, USA). The rabbit polyclonal antibody raised against adiponectin was obtained from Calbiochem (Mississaga, Ontario, Canada). The rabbit polyclonal and mouse monoclonal antibodies raised against Erk, phospho-Erk, p38 and phospho-p38 were obtained from Cell Signaling Technology Inc. (Beverly, MA, USA). The monoclonal antibody raised against fatty acid synthase (FAS) was obtained from BD Biosciences (San-Jose, CA, USA).

### Cell culture and differentiation

3T3-L1 and 293T cells were grown in Dulbecco's modified Eagle's medium (DMEM) supplemented with 10% (v/v) Bovine Calf Serum (BCS), 100 U/ml penicillin and 100 µg/ml streptomycin. To induce differentiation, two day-postconfluent cells were fed with DMEM supplemented with 10% FBS, 1 µM dexamethasone, 0.5 mM IBMX and 5 µg/ml insulin for two days. Medium was then changed for DMEM supplemented with 10% FBS and 5 µg/ml insulin. Medium was changed after two more days for DMEM supplemented with 10% FBS, and replenished every two days until harvesting. For rosiglitazone-induced activation of PPARγ, cells were induced to differentiate as stated above, with addition of 1 µM of rosiglitazone for the whole differentiation period.

### Lentivirus production and infection of 3T3-L1 cells

293T cells were cotransfected with the envelope protein expressing vector (pMD2.G, kindly provided by Dr. Didier Trono, University of Geneva Medical School, Geneva, Switzerland), the packaging protein expressing vector (psPAX2, kindly provided by Dr. Didier Trono, University of Geneva Medical School, Geneva, Switzerland) and with either the transfer pLKO.1 empty lentiviral vector (Addgene), the pLKO.1-based lentiviral mouse DLK shRNA vector (clone TRCN0000022572, Open Biosystems) or the pLKO.1-based lentiviral human DLK shRNA vector (clone TRCN0000000999, Open Biosystems) using FuGENE 6 transfection reagent (Roche Diagnostics, Laval, Québec, Canada). Briefly, cells were incubated in DMEM supplemented with 10% (v/v) FBS, antibiotics and transfection mix for 48 hours. Medium containing lentiviruses was then collected, treated with polybrene (8 µg/ml) and filtered. 24 hours prior infection, 3T3-L1 cells were seeded at a density of 2.5×10^4^ cells/ml in 100-mm dishes. The next day, medium was removed and 1 ml of lentiviral suspension was added to the plates. Cells were incubated at 37°C with the lentiviral suspension for 1 hour, and 8.5 ml of DMEM supplemented with 10% BCS and antibiotics were added. 24 hours later, cells were washed once with PBS, and either trypsinised and reseeded into four 60-mm dishes in DMEM supplemented with 10% FBS and 2 µg/ml puromycin for selection or used as is. Media was changed every 2 days until cells reached confluency, after which they were induced to differentiate as mentioned above.

### Preparation of cell or tissue lysates and immunoblotting

Cells were lysed for 60 min at 4°C in lysis buffer (50 mM Tris-HCl pH 7.4, 1% Triton X-100, 150 mM NaCl, 5 mM EDTA, 0.2 mM sodium orthovanadate, 0.2 mM sodium fluoride, 1 mM phenylmethylsulfonyl fluoride, 1 mg/ml leupeptin, and 1 mg/ml aprotinin). Lysates were clarified by centrifugation (12 000× g for 10 min at 4°C) and the concentration of total protein in the supernatant fraction was quantified by the modified Bradford protein assay (Bio-Rad Laboratories, Mississauga, Ontario, Canada). Several white and brown adipose depots provided by the animal facility of our Department were collected from female CD-1 mice. For preparation of the homogenates, the tissues were washed with ice-cold phosphate-buffered saline after their removal, quickly frozen in liquid nitrogen and ground to a fine powder with a mortar and a pestle. The tissue powder was then resuspended in the cell lysis buffer described above, incubated for 60 min at 4°C and clarified by centrifugation. Quantification of the tissue lysates was also done by the modified Bradford protein assay. For immunoblotting, equal amounts of proteins were fractionated by SDS-polyacrylamide gel electrophoresis (PAGE) and transferred onto polyvinylidene difluoride (PVDF) membranes (Roche Diagnostics, Laval, Québec, Canada) using a semidry transfer apparatus (Bio-Rad Laboratories, Mississauga, Ontario, Canada). Membranes were incubated overnight on a rotating plate at 4°C in a solution containing 20 mM Tris, pH 7.5, 150 mM NaCl, 0.1% Tween-20 (TBS-T) supplemented with 5% skim milk powder (w/v) and the primary antibody. Membranes were washed two times in TBS-T before incubation in a solution containing TBS-T, 5% skim milk powder (w/v) and the secondary horseradish peroxidase-conjugated antibodies on a rotating plate for 1 hour at room temperature. Membranes were washed two more times in TBS-T before immunoreactive bands were detected by enhanced chemiluminescence (ECL Plus Western blotting kit, Amersham Pharmacia Biotech, Inc.).

### Quantification and staining of lipids with Oil Red O

Treated cells were carefully washed two times with PBS at room temperature and then fixed for 30 min with 10% formaldehyde in PBS. Each dish was then rinsed three times with distilled water. Lipid droplets were stained for 15 min at room temperature with a freshly made and filtered working solution of 0,3% Oil Red O. Cells were then washed once with 70% ethanol, twice with distilled water and photographed. Lipid quantification was done by incubating stained cells with gentle agitation for 5 min in a 4% (v/v) solution of NP40 in isopropanol. Supernatant was then analysed with a spectrophotometer at 520 nm.

### Immunocomplex kinase assay for DLK

3T3-L1 cells at 0, 2, 4, 6, 8 or 10 days of differentiation were homogenized in lysis buffer (50 mM Tris-HCl pH 7.4, 1% Triton X-100, 150 mM NaCl, 5 mM EDTA, 0.2 mM sodium orthovanadate, 0.2 mM sodium fluoride, 1 mM phenylmethylsulfonyl fluoride, 1 mg/ml leupeptin, and 1 mg/ml aprotinin). Lysates were clarified by centrifugation and the concentration of total protein in the supernatant fraction was quantified using the modified Bradford protein assay (Bio-Rad Laboratories). Typically, 500 µg of protein extract were incubated overnight at 4°C with constant rotation using DLK polyclonal antibody (Abgent, 1∶100 dilution) and protein A–sepharose beads. After incubation, the immunocomplexes were washed three times with lysis buffer and three times with kinase buffer (10 mM Tris-HCl pH 7.4, 150 mM NaCl, 10 mM MgCl2, 0.5 mM DTT, 0.1 mM phenylmethylsulfonyl fluoride, 0.2 mM sodium orthovanadate, 1 mg/ml leupeptin, 1 mg/ml aprotinin). Immunocomplex kinase assays were performed by incubating the immune complexes in 40 µl of kinase buffer containing 2.5 mCi of [γ^32^P]ATP (Amersham Pharmacia Biotech Inc.), 25 mM ATP, and 1 mg of myelin basic protein as substrate. Following 20 min incubation at 30°C, the reaction was stopped by adding an appropriate volume of 6× SDS-PAGE sample buffer and boiling for 5 min. Phosphorylated proteins were visualized by autoradiography after fractionation by SDS-PAGE.

### Chromatin immunoprecipitation

Each experiment was done with one confuent 100-mm dish of 2 days differentiated 3T3-L1 cells infected with an empty lentiviral vector (Empty vector) or lentiviruses expressing mouse (mDLK) or human (hDLK) shRNA. Briefly, infected cells were crosslinked for 10 min at room temperature with 1% formaldehyde in PBS. Cells were then washed in PBS, resuspended in 200 µl of ChIP lysis buffer [1% sodium dodecyl sulfate (SDS), 10 mM EDTA, 50 mM Tris-HCl (pH 8.0), and protease inhibitors] and sonicated (60% maximum output for 15 s with 3 min pause, 3 cycles; Branson Sonifier Type 450 with microtip; Danbury, CT) in an ice bath. The chromatin solution was diluted 10-fold in ChIP dilution buffer (0.01% SDS; 1.1% Triton X-100; 1.2 mM EDTA; 16.7 mM Tris, pH 8.1; 16.7 mM NaCl; and protease inhibitors). 5% of the lysate was used for purification of total DNA. Each sample was precleared by incubating with 2 µg salmon sperm DNA/protein A-agarose 50% gel slurry (Roche Diagnostics, Laval, Quebec, Canada) for 2 hours at 4°C. An aliquot of 10 µg of indicated antibody (or no antibody for the control) was added and immunoprecipitated at 4°C overnight. The immunoprecipitate was collected using salmon sperm DNA/protein A-agarose and washed sequentially with the following buffers: low-salt wash buffer (0.1% SDS; 1% Triton X-100; 2 mM EDTA; 20 mM Tri-HCl, pH 8.1; 150 mM NaCl); high-salt wash buffer (0.1% SDS; 1% Triton X-100; 2 mM EDTA; 20 mM Tris-HCl, pH 8.1; 500 mM NaCl); LiCl wash buffer (0.25 M LiCl; 1% Nonidet P-40; 1% sodium deoxycholate; 1 mM EDTA; 10 mM Tris-HCl, pH 8.1) and TE (10 mM Tris-HCl, pH 8.0; 1 mM EDTA). DNA- protein cross-links were reversed by incubation at 65°C overnight followed by proteinase K treatment. DNA was recovered by purification with the Qiaquik PCR purification column (Qiagen). Results were analysed by real-time PCR with primers spanning C/EBPβ binding site at the C/EBPα (*forward*: 5′-TAGTGTTGGCTGGAAGTGGGTGACTTAGAGGC-3′, *reverse*: 5′-TTCTCCTGTGACTTTCCAAGGCGGTGAGTG-3′) and PPARγ2 (*forward*: 5′-TACGTTTATCGGTGTTTCAT-3′, *reverse*: 5′-TCTCGCCAGTGACCC-3′) promoters.

### Real-time quantitative reverse transcription PCR

Total RNA was extracted using Trizol reagent (Invitrogen, Burlington, Ontario, Canada). Reverse transcription was done on total RNA with random hexamers as primers and the Moloney murine leukemia virus reverse transcriptase (Promega, Madison, Wisconsin, USA). Quantitative Real Time PCR reactions were run on an ABI 7500 (Applied Biosystems) apparatus and results were analysed with SDS software (Applied Biosystems). Amplification patterns were normalized on the 36B4 housekeeping gene. The primer used for amplification were: *forward*: 5′-CGACCTGGAAGTCCAACTAC-3′ and *reverse*: 5′-ATCTGCTGCATCTGCTTG-3′ for 36B4, *forward*: 5′-GGACAAGCTGAGCGACGAGTA-3′ and *reverse*: 5′-CCGTCAGCTCCAGCACCTT-3′ for C/EBPβ, *forward*: 5′-GCGCAAGAGCCGAGATAAAG-3′ and *reverse*: 5′-CACGGCTCAGCTGTTCCA-3′ for C/EBPα, *forward*: 5′-GCCCAGGCTTGCTGAACGTGAAG-3′ and *reverse*: 5′-CACGTGCTCTGTGACGATCTGCC-3′ for PPARγ, *forward*: 5′-CATCCCAGGACATCCTGGCCACAATG-3′ and *reverse*: 5′-GGCCCTTCAGCTCCTGTCATTCCAAC-3′ for adiponectin and *forward*: 5′-GCTATGCAGATGGCTGTCTCTCCCAG-3′ and *reverse*: 5′-GCAGCGCTGTTTACATTCCTCCCAGG-3′ for fatty acid synthase.
